# The Interactive Roles of *Aedes aegypti* Super-Production and Human Density in Dengue Transmission

**DOI:** 10.1371/journal.pntd.0001799

**Published:** 2012-08-28

**Authors:** Harish Padmanabha, David Durham, Fabio Correa, Maria Diuk-Wasser, Alison Galvani

**Affiliations:** 1 Yale School of Public Health, New Haven, Connecticut, United States of America; 2 Instituto Nacional de Salud, Bogota, Colombia; University of Texas Medical Branch, United States of America

## Abstract

**Background:**

*A. aegypti* production and human density may vary considerably in dengue endemic areas. Understanding how interactions between these factors influence the risk of transmission could improve the effectiveness of the allocation of vector control resources. To evaluate the combined impacts of variation in *A. aegypti* production and human density we integrated field data with simulation modeling.

**Methodology/Principal Findings:**

Using data from seven censuses of *A. aegypti* pupae (2007–2009) and from demographic surveys, we developed an agent-based transmission model of the dengue transmission cycle across houses in 16 dengue-endemic urban ‘patches’ (1–3 city blocks each) of Armenia, Colombia. Our field data showed that 92% of pupae concentrated in only 5% of houses, defined as *super-producers*. Average secondary infections (R_0_) depended on infrequent, but highly explosive transmission events. These s*uper-spreading* events occurred almost exclusively when the introduced infectious person infected mosquitoes that were produced in super-productive containers. Increased human density favored R_0_, and when the likelihood of human introduction of virus was incorporated into risk, a strong interaction arose between vector production and human density. Simulated intervention of super-productive containers was substantially more effective in reducing dengue risk at higher human densities.

**Significance/Conclusions:**

These results show significant interactions between human population density and the natural regulatory pattern of *A. aegypti* in the dynamics of dengue transmission. The large epidemiological significance of super-productive containers suggests that they have the potential to influence dengue viral adaptation to mosquitoes. Human population density plays a major role in dengue transmission, due to its potential impact on human-*A. aegypti* contact, both within a person's home and when visiting others. The large variation in population density within typical dengue endemic cities suggests that it should be a major consideration in dengue control policy.

## Author Summary

In the urban dengue system the life history of the mosquito vector, *Aedes aegypti*, transpires mainly inside and around human residences. In this study we integrated field data from an endemic city of Colombia into a simulation model to assess how natural variation in *A. aegypti* production and household human density influence dengue transmission. Contrary to traditional models, we show that the basic reproductive rate of dengue (R_o_) is more likely to be positively correlated with human density. Moreover, the natural regulatory pattern of *A. aegypti* production, where a few *super-productive* houses dominate vector recruitment, caused a "super-spreading" pattern, whereby the large majority of viral introductions did not generate secondary infections, and R_o_ depended on sporadic, highly explosive transmission events. These events were dependent on the introduced infectious human infecting mosquitoes produced in super-productive vessels. When the likelihood of human introduction was incorporated into our risk indicator, a significant interaction emerged between human density and *A. aegypti* super production, such that removal of these containers had a much larger impact on reducing dengue in areas of higher human density. These results show that knowledge of interactions between human population density, social interactions and the natural regulatory pattern of *A. aegypti* can improve the design of dengue control efforts.

## Introduction

In the latter half of the 20th century dengue emerged as the most prevalent urban vector borne disease of humans, readily propagating among urban populations of humans and *Aedes aegypti* mosquitoes. Intervention against *A. aegypti* domestic container habitats, known as source reduction, is central to the dengue prevention activities of most health departments of endemic cities [1]–[4]. However, few programs have the resources necessary to intervene effectively in all areas infested by *A. aegypti* [5]. Therefore, dengue prevention could benefit from an understanding of the areas in which the impact of source reduction would be maximized.

Most evidence indicates that urban *A. aegypti* populations are regulated by mortality that occurs in the egg/larval stages [6]–[9]. This results in the common finding that most infested containers produce few pupae, whereas the majority of the adult vector population derives from only a few containers and houses, called *super-producers* [4], [8], [10], [11]. The elimination of super-productive containers forms the conceptual basis for targeting source reduction programs [2], [3], [12]. This approach is grounded in modeling studies that show that the targeted elimination of containers above a threshold pupal abundance can significantly reduce the risk of dengue [13], [14]. However, this consistent regulatory pattern of *A. aegypti* also causes the majority of vectors to emerge in the same location, generating significant spatial heterogeneity in adult vector distributions [15]–[18]. However, because most models of the impact of source reduction on dengue assume homogenous mixing between humans and mosquitoes, little is known about how the phenomenon super-production *per se* affects transmission dynamics. This knowledge is critical to understanding how the natural regulation of *A. aegypti* influences the dynamics of dengue.

Traditionally, field estimates of the entomological risk of mosquito-borne disease have focused on the ratio of vectors to humans, in order to estimate the rate at which humans receive infectious bites [19]. This rationale has been used to assess the entomological risk of dengue through surveys of human and pupal abundance in order to estimate the metric *A. aegypti* pupae per person [14]. Unlike other mosquito-human systems *A. aegypti* rests, feeds and oviposits largely inside houses, generating a close physical proximity to humans. This co-habitation likely also explains why *A. aegypti*, exhibits adaptations such as asynchronous ovarian development and the preferential use of human blood rather than sugar as an energy sourcefor reproduction [20]–[22]. Consequently, female *A. aegypti* repeatedly bite humans even when seeking to lay eggs. This differs from other mosquito disease systems with a larger distance between blood-seeking and oviposition sites, in which host-vector contact is strongly determined by the time required for the mosquito to process the blood meal, oviposit and once again encounter hosts [19]. Greater human density may therefore increase the number of humans that each *A. aegypti* encounters, a process that is overlooked when risk is measured by the vector to host ratio. Because few targeted source reduction programs consider heterogeneities in human density when determining geographic targets, improved knowledge of the combined epidemiological impacts of super-productive vessels and human density can play an important role in optimizing dengue surveillance and control.

A second important feature of the urban dengue system is the capacity of human movement across urban areas to propagate infection spatially [23]–[26]. As human density increases, so do the number of different people that a person encounters, increasing both the number of mosquitoes that potentially bite a person and the number of unique people that a mosquito may bite. Moreover, because each person has a unique social contact network, greater human density in a particular area will increase the frequency of dengue infected visitors. Therefore, human density may influence the risk of dengue epidemics in a given area by affecting both the average number of secondary cases (R_o_) and the frequency of viral introduction. As our measure of risk, we used the average secondary human infection rate for a given per-capita rate of dengue introduction into an immunologically naïve population [27], an index that we term epidemic potential. Because both the rates of secondary infection and viral introduction may increase with human density, epidemic potential captures the human density-dependence of dengue risk better than R_0_ alone.

Human and mosquito population sizes influence dengue transmission by two distinct processes: (1) human transmission to mosquitoes and (2) mosquito transmission to humans [28]. The first depends on the number of unique mosquitoes that bite each person, whereas the second is determined by the number of unique people that each infected mosquito bites. A greater human density may decrease the number of mosquito bites received by each person but increase the number of people that each mosquito bites, complicating efforts to estimate dengue risk for a given population. Variation in the rate of vector production will directly impact human transmission to mosquitoes, but only indirectly affect mosquito transmission to humans through the abundance of infectious mosquitoes.

Additionally, the characteristic aggregation of *A. aegypti* across houses suggests a low probability of a high-impact event. That is, if an infectious person contacts a house where mosquitoes aggregate, many potentially infected mosquitoes may result. However, when mosquitoes are aggregated in only a few houses, it is more likely that a randomly introduced human infection will contact a house with few mosquitoes, resulting in a small number of secondary cases. Understanding the balance between human density and these opposing influences of mosquito aggregation is essential for entomological risk assessment and for the optimization of source reduction strategies.

In this paper we integrate field data with simulation modeling in order to develop a better understanding of how the interaction between human and mosquito densities facilitates dengue transmission and to provide guidelines for designing and evaluating targeted prevention programs. Using field-collected snapshots of the distribution of *A. aegypti* pupae and humans across houses surveyed in Armenia, Colombia, we evaluated the impact of human and *A. aegypti* pupal densities on the simulated number of secondary human infections and the epidemic potential. We determined how *A. aegypti* production and human densities affected the propagation of dengue across houses and identified the field indices that most correlate with entomological risk. In addition, we used a vector control simulation to determine how human density modulates the long-term impact of targeted control of highly productive *A. aegypti* habitats.

## Materials and Methods

### Field data

**Figure 1:**
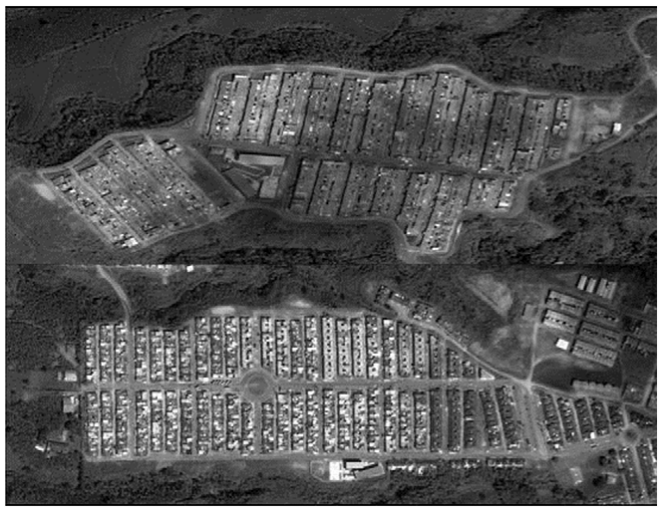
Neighborhoods of study in the city of Armenia, Colombia.

The field data used in this study was collected in Las Colinas and La Fachada, two highly endemic neighborhoods of the city of Armenia, Colombia. Armenia had the highest number of reported dengue cases of any Colombian city between 2001 and 2008, and the highest cumulative incidence between 2001 and 2011, according to surveillance records of the Instituto Nacional de Salud (INS) of the Colombian Ministry of Health. La Fachada and Las Colinas have elevations of 1335 and 1329 m, respectively and we have observed mean ambient temperatures of 24–25°C through limited surveillance using thermal sensors. In each neighborhood (Fig. 1) we randomly selected eight study patches comprising between 41 and 142 houses (1–3 adjacent blocks) for a total of 16 patches across the city. Over a 26-month period seven censuses of water-holding vessels, including counting of all *A. aegypti* pupae, were conducted in each patch by public vector control technicians under supervision of Armenia's Health Secretary and INS investigators. This provided us with a dataset of 112 sample distributions of mosquito pupae and humans (seven surveys for each of the 16 patches), with minimal variation in the types of productive containers, housing structure, climate, housing density, and vegetation across samples. An exhaustive human demographic census was conducted in 2009.

### Model description

#### Overview

Our model simulates dengue transmission across houses arranged in 16 different grids, each representing one of the study patches. Mosquitoes randomly bite humans in their residence and may change residences between time-steps. Humans live in fixed residences, but visit other houses where they are potentially exposed to mosquito bites. The entrance of host-seeking mosquitoes is derived from pupal counts, and each mosquito has an independent daily probability of survival *s_v_*. Human populations and spatial distribution are derived from census data and are fixed, with each person assigned an independent probability of introducing an infection from outside the patch. The model tracks susceptible, exposed, and infectious humans and mosquitoes in an immunologically naïve patch. Viral introduction and tracking of infection status begins when mosquito populations reach a spatiotemporal equilibrium that spans ∼15–25 days, the time required for the virus to complete a single transmission cycle (human-to-mosquito and mosquito-to-human). The simulation ceases when all mosquitoes that were infected by the introduced human infection die.

#### Simulation grids

The number, size, and spatial layout of houses in each grid were derived from urban planning maps of the study patches obtained from the Armenia Municipal Planning Department. Most premises are small, averaging 44 m^2^ and 38 m^2^ in Las Colinas and La Fachada, respectively, with little variation between houses (Fig. 1). In our model the dimensions of each house are assumed to be 5 m×8 m, approximating the average dimensions of a lot in our study neighborhoods. We made minor alterations to the layout of the field patches while keeping the layout as close as possible to reality. These alterations ensured that all simulations contained 2–4 parallel columns of houses for model tractability. In addition, we surrounded each simulated patch by a buffer zone of two houses that provided a potential refuge for emigrating mosquitoes and a source of immigrant mosquitoes. By allowing mosquitoes to move in and out of the patch, we approximated the effect of adjacent blocks without explicitly modeling them. The model tracked production, death and dispersal of mosquitoes, but not the infection status or the people in the buffer zone. The combination of the buffer zone and minor changes in the alignment of houses allowed us to isolate the effects of vector production on dengue transmission better, independent of potential interactions with block level landscape features and adjacent blocks.

#### Vector production and human density

Given the short duration of the simulation, the number of human residents and the mean rate of vector production in each house are assumed constant and are parameterized from field data. The number of pupae in a house that could not be inspected in a particular survey was extrapolated based the house's infestation frequency in other surveys and the distribution of pupae across infested vessels (see Text S1 for details).The mean daily recruitment rate of host-seeking vectors in each premise is modeled by a Poisson distribution with parameter 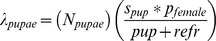. Here, *N_pupae_* is the field-observed number of pupae in each home survey, *p_female_* is the probability of a mosquito being a female (0.5). *s_pup_*, the probability of a pupae surviving to maturity and *pup*, the temperature-dependent duration of the pupal stage (2.7 days) were both measured in semi-field conditions in Armenia (Padmanabha, unpublished data); *refr* is the temperature-dependent time that female *A. aegypti* are not receptive to mating and therefore do not host-seek (assumed 3 days at 22°C, based on [29]). Mosquitoes can potentially disperse from the house containing their larval habitat two days after hatching (Table S1).

#### Vector survival and dispersal

Parameter ranges used for vector survival and movement are based on mark-release-recapture studies conducted in a dengue endemic *favela* of Rio de Janeiro [30], with housing density and social characteristics similar to those of our study neighborhoods in Armenia. For simplicity, we treat the daily probability of mosquito survival (*s_v_*) as age-independent. Each mosquito starts the day in the center of the property in which it is located, based on indoor resting behavior of *A. aegypti* [31]. Mosquitoes may randomly disperse across houses, moving in accordance with a two-dimensional Normal distribution with mean 0 and standard deviation *σ_mos_*. The range of the dispersal parameter (*σ_mos_*) was determined by simulating the daily fraction of females that changed houses, and calibrated to the range estimated in [30].

#### Vector biting

We assume that mosquitoes randomly feed on all humans located within the same house in which they begin each time step, with probability of becoming satisfied *p_full_*. Mosquitoes continue to bite randomly until they either become satisfied or have bitten a total of π times. We allow mosquitoes to sample p_full_ from the range 0.25–0.5 per day (2–4 attempted bites per day), and specified π at 10, comparable to previous models [32].

#### Human movement

Previous studies demonstrated the importance of incorporating human movement patterns into dengue transmission models [23]–[25]. In our model, each person makes one visit per day in which they are potentially exposed to *A. aegypti*. The location of the visit is chosen randomly from a fixed set of contact premises that are defined at the start of each model iteration. We assume that human visits that entail exposure to biting *A. aegypti* are more likely to occur in closer neighbors. For example, in our extensive qualitative and quantitative field work in Las Colinas and La Fachada [8], we have observed that children that live on the same street enter each others' house. Thus, for each premise, the probability of falling into the fixed contact set of a non-resident person is the sum of a distance-independent component, *pIndep*, and a distance-dependent component, *Cd*. In the distance-dependent component, the probability that a house on the same street falls into the contact set of an individual is proportional to the inverse of the square root of the distance between that house and the house of the individual. In the absence of data on which types of visits expose people to *A. aegypti* bites, we specify both *Cd* and *pIndep* at 0.2. This approach to modeling human movement guarantees that while the rate of visiting remains constant for each patch, the number of houses that a person might potentially visit increases with patch size.

Each blood-seeking mosquito encounters either resident or visiting humans, randomly choosing between them. To incorporate the differential exposure of residents and visitors, when a mosquito selects a person to bite it does so with a weight of *visit*W for visitors and 1-*visit*W for residents. Because *A. aegypti* bites occur during early morning hours and in areas on the interior of houses [31], such as bedrooms, in which most visitors are less likely to frequent, we specify *visit*W as 0.2, such that each resident is four times more likely than each visitor to be bitten by local *A. aegypti*.

#### Viral introduction

Because of the short lifespan and dispersal range of *A. aegypti* [30], the short infectious viremia in humans and the limited availability of susceptible humans, dengue cannot persist in small areas such as a neighborhood without a continuous source of human infection from external areas [23]. Accordingly, we modeled viral introduction only through infectious humans. Each person in the simulation is assigned an independent daily probability of becoming viremic, *pintro*. This allowed us to incorporate viral introduction into the analysis of the number of people in the patch. To focus the analysis on the spread of a single introduction, viral introduction ceases when at least one dengue case appears.

### Analyses

We conducted three separate analyses using our model. First, to evaluate the effects of host densities on the generation of secondary cases we ran the model for 300 iterations for each of the 112 patch-surveys (33,600 total iterations), while introducing a single infectious human in every iteration. Second, to simulate dengue introduction from an external area we again ran the model across the range of scenarios while stochastically introducing dengue by assigning a probability of becoming infectious (*intro*) to each person. Finally, to evaluate targeted vector control interventions in variable human densities, we simulated the elimination of all containers containing a threshold number of pupae in each of the 16 patches, while varying human density by a fixed proportion of the field-observed value.

#### Secondary infections

We analyzed variation across iterations in order to explore how the introduced infections propagated across houses. We determined the number of mosquitoes exposed by each index human viremia and the average number of human exposures per infectious mosquito. We then evaluated how the number of co-habiting pupae in a house affected the rate at which a mosquito received infection from humans (human to mosquito spreading) or transmitted to humans (mosquito to human spreading). Humans density was also compared with the rate of each type of transmission event. To evaluate the overall effects of these patterns on transmission intensity, we used a multinomial negative binomial model (STATA 10) of the number of secondary infections. Five covariates were included: total human population (in the patch), humans per house, average pupae per house, number of people in the *I_0_* house, and number of biting mosquitoes in the *I_0_* house. A categorical patch-survey variable, grouping across iterations for each survey of each patch, was included to account for variation due to the specific spatio-temporal conditions of the patch surveys not explained by the other covariates.

#### Epidemic potential

Upon assigning a per-capita daily probability of human introduction of virus, we evaluated the overall rate of secondary infections (epidemic potential) . In eachrun stochastic introduction was disabled once the first infection appeared. The extremely rare iterations in which more than one infection was introduced on the same day were excluded from the analysis.

Univariate regressions were used to evaluate the utility of different entomological surveillance indices for assessing epidemic potential. We employed a generalized least squares (GLS) regression that took into account both the repeated measurements (surveys) in each patch and heteroskedasticity in the distribution of risk across patches. The following indices were evaluated using log-likelihoods and test-statistics of their respective univariate model: 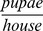, 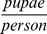, 
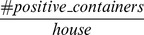
 (Breteau Index), 
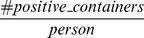
, 
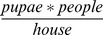
 , 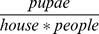.

#### Control simulation

To investigate the effects of the distribution of mosquito production and human density on dengue control, we simulated the targeted elimination of vector production in households with a threshold number of pupae, while varying the human population in each patch. To incorporate the effects of long-term variation in vector production, for each iteration of a patch we randomly chose one of the field surveys to parameterize the pupal distribution. 1000 vector control iterations were simulated. In order to evaluate the effect of targeted control of a subset of highly productive houses on epidemic risk, we first parameterized the pupae in each house from the field data, and then removed all pupae in houses above a threshold pupal count. We varied this control threshold on a logarithmic scale from 1 to 1000 pupae in order to evaluate a range of both highly focused and extensive control strategies and determine the minimum household pupal threshold that source reduction programs should target. To determine whether human density influences the impact of vector control, we simultaneously varied the human population in each patch from 50% to 100% in intervals of 5% of the observed human density. This approach accounted for potential colinearities between vector production and human density in the field data. As both neighborhoods studied are among the highest population densities in Armenia, we restricted our focus to reduction in human density.

## Results

**Figure 2:**
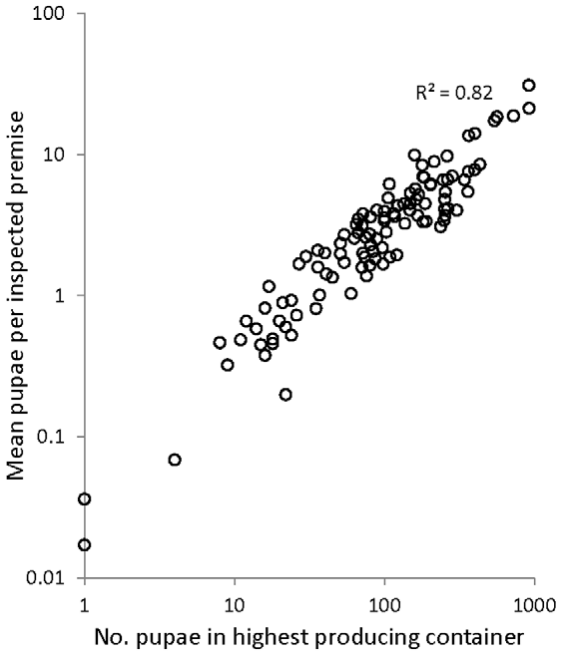
Relationship between pupal abundance in highest producing container and average pupae per premise across 112 patch-surveys in Armenia.

Over the seven surveys, we found 1,707 vessels infested with *A. aegypti* and counted a total of 32,058 pupae. Inspection percentage of the 1364 premises in the study patches averaged 70.4% across the seven surveys, with 79% of all premises inspected in at least four of the seven surveys. Our 16 surveyed patches ranged from 170 to 587 people and from 41 to 142 houses. Human densities in study patches ranged from 3.2 to 4.5 residents per house (Table 1). Over the 112 patch-surveys (7 surveys of each patch), the mean number of *A*. *aegypti* pupae per house ranged from 0.017 to 30.9. Pupal production was highly aggregated, with 92% of the total pupae found in only 5% of the house-surveys (those containing at least 16 pupae). Roughly 80% of the variation in vector production could be explained by pupal abundance in the most productive container in each of the 112 patch-surveys (Fig. 2).

**Table 1:** Host density input data and simulated basic reproductive rate (*R*_0_, number of secondary human infections averaged across 300 model iterations for each of 7 separate pupal surveys of dengue virus for each study patch.

Residents	Houses	Residents per house	Total *A. aegypti* Pupae	Avg. pupae per house per survey (SD across surveys)	*R*_0_ (percent of iterations with ≥1 secondary infection)
170	41	4.1	1787	6.2 (9.8)	2.85 (22%)
184	58	3.2	282	0.7 (0.7)	0.88 (13%)
209	50	4.2	780	2.2 (1.6)	1.25 (16%)
226	55	4.1	1152	3.0 (2.3)	1.66 (19%)
256	62	4.1	2292	5.3 (2.6)	2.23 (31%)
259	74	3.5	584	1.1 (0.8)	0.92 (12%)
282	69	4.1	2944	6.1 (6.9)	2.59 (28%)
312	69	4.5	2145	4.4 (2.1)	1.90 (27%)
317	74	4.3	3876	7.5 (7.5)	2.70 (25%)
338	80	4.2	3292	5.9 (4.8)	2.60 (31%)
342	83	4.1	1044	1.8 (1.4)	1.54 (16%)
397	99	4.0	2800	4.0 (1.0)	2.89 (28%)
400	103	3.9	2069	2.9 (2.5)	1.97 (21%)
405	90	4.5	4433	7.0 (6.3)	3.57 (30%)
428	102	4.2	1870	2.6 (1.1)	1.67 (22%)
587	142	4.1	7419	7.5 (4.0)	3.87 (34%)

**Figure 3:**
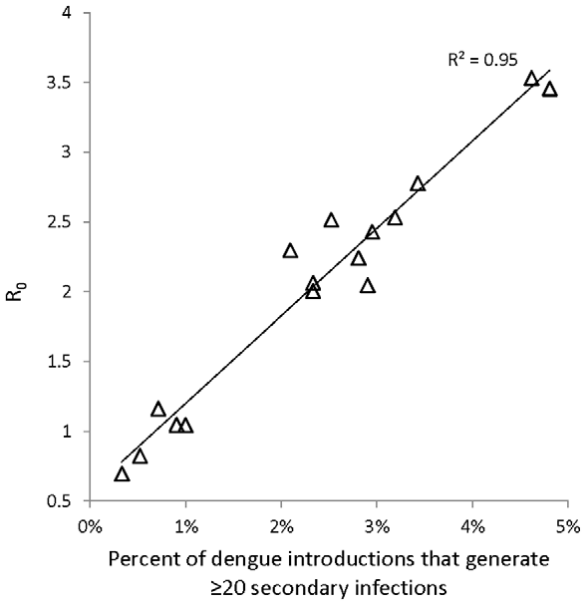
Correlation between percent of dengue introduction iterations that generate ≥20 secondary infections and R_0_ (secondary infections averaged over 300 introduction trials for each of 7 pupal surveys) across 16 study patches in Armenia, Colombia.

Across our simulations, 72% of the 112 patch-survey scenarios had *R_0_*>1 (average number of secondary infections across 300 model iterations). R_0_ across patches (secondary infections averaged across all iterations of all seven surveys) varied between 0.88 and 3.87, and was above 1 in 14 of 16 patches (Table 1) Variation in R_0_ across patches was highly correlated to the frequency of viral introductions that generated greater than 20 secondary infections (R^2^ = 0.95, Fig. 3), which represented only 10 percent of the total model iterations. In all patches the large majority of viral introductions did not result in secondary transmission (Table 1). This indicates that secondary transmission was largely driven by the occurrence of highly explosive transmission events.

### Determinants of secondary infections

**Figure 4:**
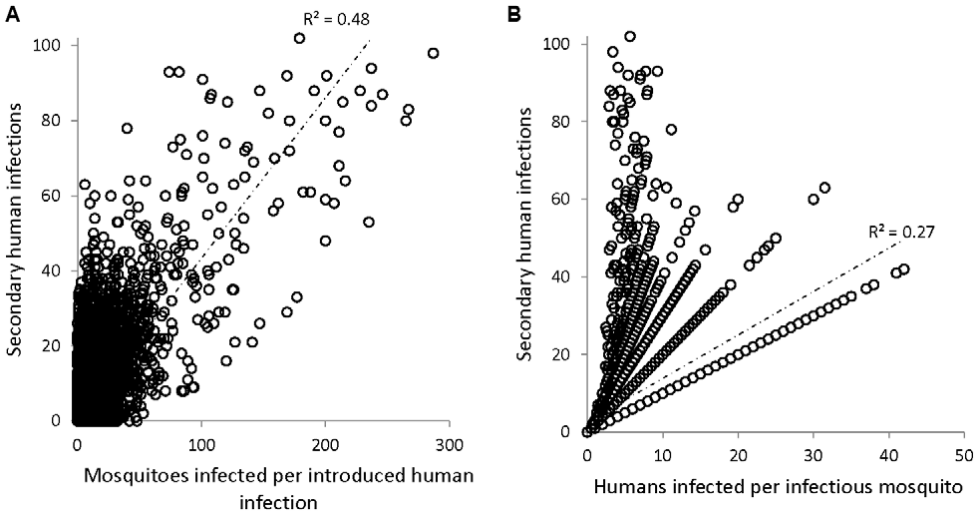
Effects of each type of transmission event on secondary infections. (a) Correlation between human-to-mosquito transmission (average number of infected mosquitoes per human introduction) and secondary human infections and (b) Correlation between mosquito-to-human transmission (average number of humans infected per infectious mosquito) and secondary human infections.

**Figure 5:**
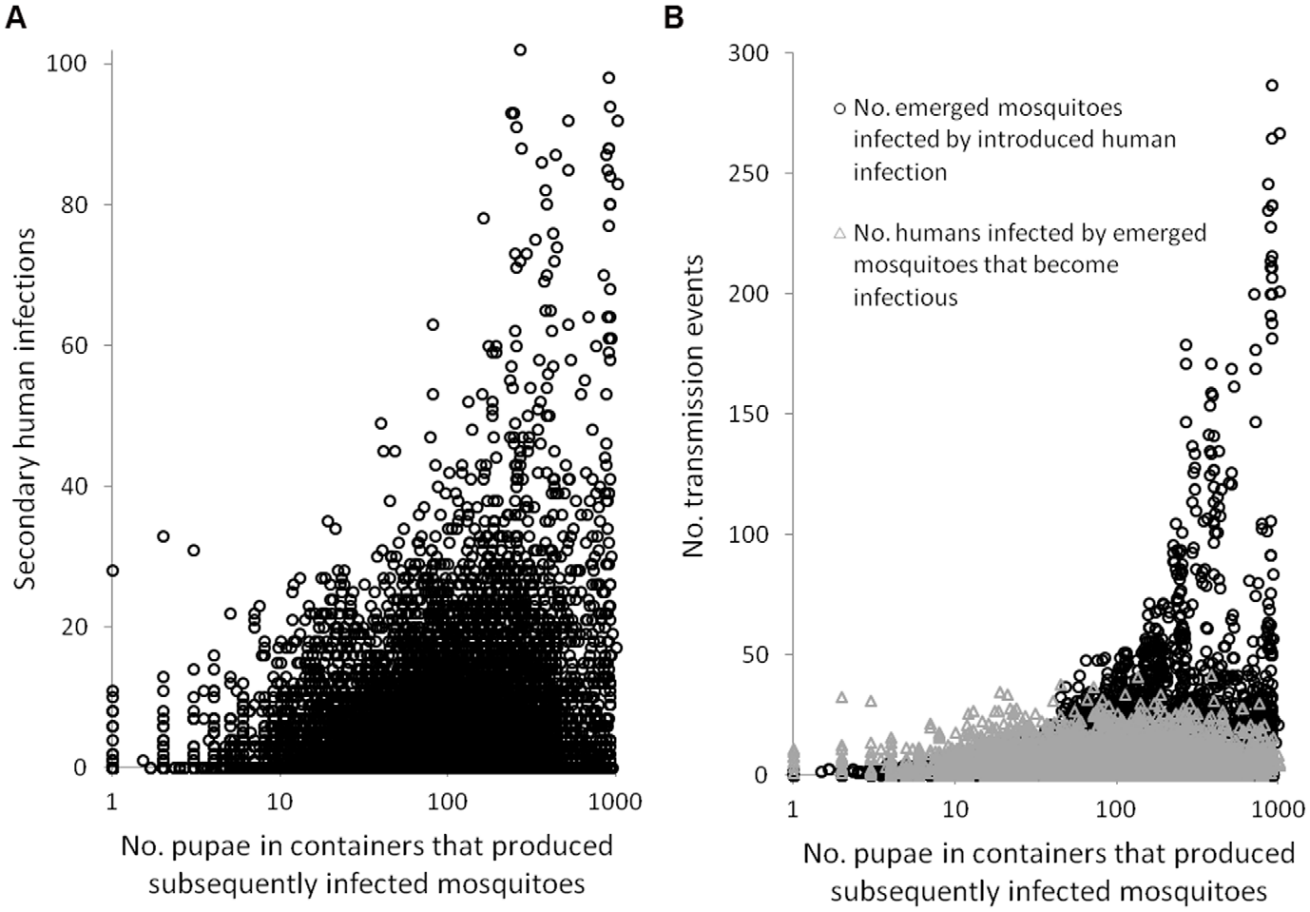
Pupae per container and transmission in subsequently emerged mosquitoes. (a) Effect of pupal abundance on human-to-mosquito infection (no. subsequently emerged mosquitoes infected by introduced human infection) and mosquito-to-human infection (avg. number of. humans infected by subsequently emerged mosquitoes that became infected); (b) Effect of pupal abundance in containers that produce infected mosquitoes on avg. number of secondary infections.

We correlated human-to-mosquito and mosquito-to-human transmission with secondary infections across each of the 33,600 iterations (300 iterations for each of the 112 patch-surveys). Human-to-mosquito transmission explained roughly 20% more of the variation in secondary infections (Fig. 4a) than mosquito-to-human transmission (Fig. 4b). Many introductions that resulted in highly explosive transmission occurred when infectious mosquitoes transmitted to relatively few humans (Fig. 4b). We used the model to evaluate pupal abundance in containers that produced infected mosquitoes. Introduced human infections could only infect large number of mosquitoes when these were produced in containers with high pupal abundance (Fig. 5a). For example, in 10% of model iterations (with at least one infected mosquito) the introduced human infection infected greater than 10 mosquitoes. These mosquitoes were *never* produced in containers with a mean pupal count less than 16 (corresponding to less than 5% of house-surveys) (Fig. 5a). By contrast, transmission to humans per infectious mosquito was not associated with the number of pupae in the containers that produced the infectious mosquitoes. When infected mosquitoes were produced in containers with few pupae, very few model iterations produced high levels of secondary human infection (Fig. 5b). For example, infected mosquitoes generated ≥20 secondary human infections in only 0.4% of the trials in which they were produced in containers with on average less than 16 pupae.

**Figure 6:**
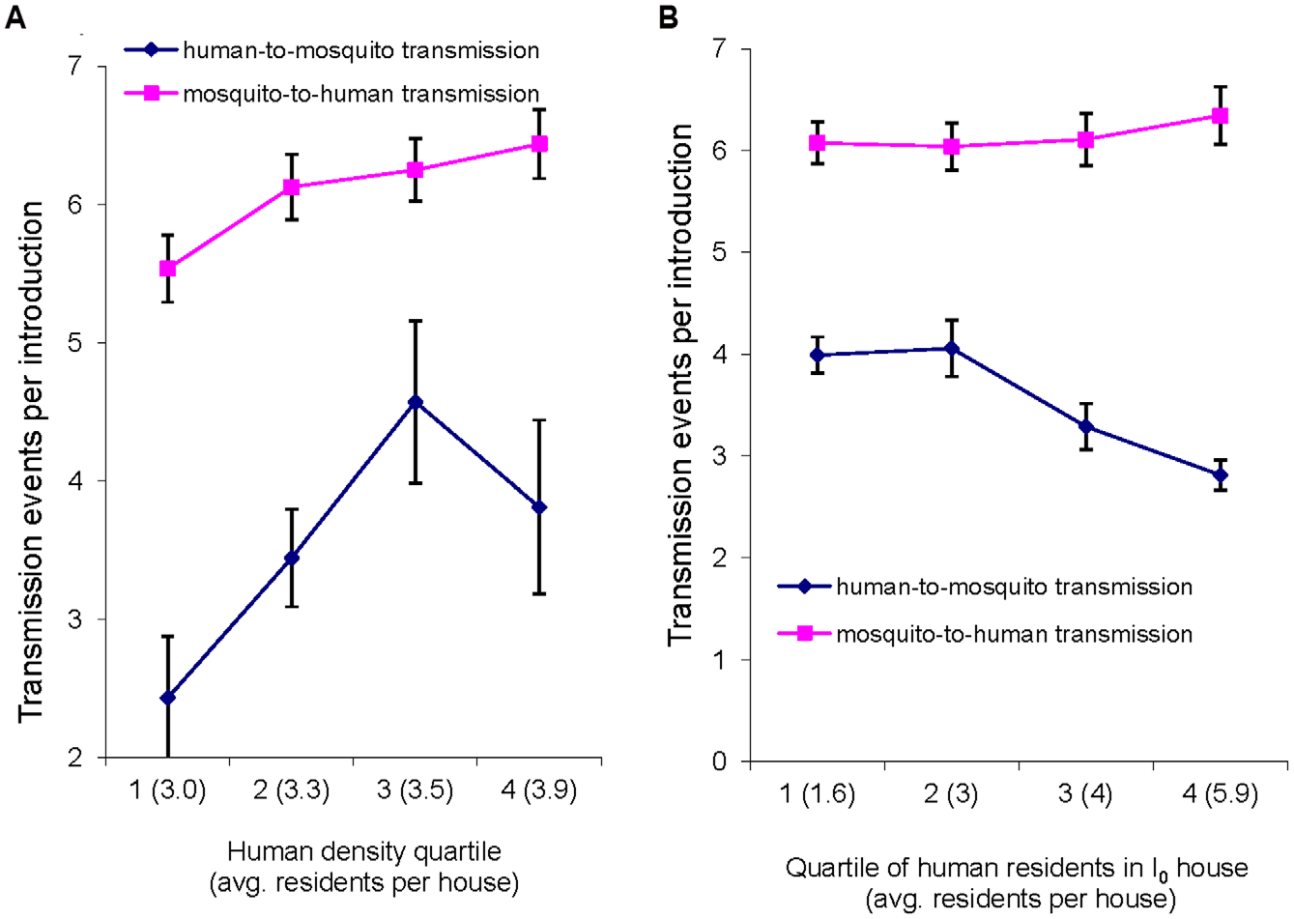
Human density and transmission events (mosquito-to-human and human-to-mosquito transmission). (a) Effects of patch-wide human density (total residents divided by houses); (b) Effects of density of residents in the house of the initial human infection (*I_0_*). Error bars are 95% CIs.

Because of model stochasticity and limited variability in human density across patches, human density effects were analyzed across quartiles of patch-wide human density (Fig. 6a) and the density of residents in the *I_0_* house (Fig. 6b). Both mosquito-to-human and human-to-mosquito transmission were significantly lower in density quartile 1 (Fig. 6a). By contrast, increased number of residents in the *I_o_* house had little impact on mosquito to human spreading, but significantly reduced human to mosquito spreading as resident density increased between quartiles 2 and 4.

A multinomial negative binomial model revealed that the number of humans per house, the average daily number of biting vectors in the *I_0_* house, and the total number of pupae all increased dengue transmission, while the number of resident humans in the *I_o_* house negatively affected transmission (Table 2). Secondary infections were more closely associated with *I_0_* vectors (Z = 28.3) than with total pupae (Z = 15.0). Total human population size did not significantly affect transmission when the other covariates were included (Table 2). These results suggest that the aggregated distribution of pupae may cause explosive dengue transmission through the concentration of biting mosquitoes in the *I_0_* house. They also suggest that although increased human residents dilute each individual contact with mosquitoes at the household level, transmission across houses at the patch level is favored by human density. Overall, these data show that vector production and human density can influence the dengue R_0_ through both household and community level processes.

**Table 2:** Multivariate negative binomial model of secondary human infections (n = 33,600 viral introductions).

Covariate	Coefficient	CI	Z	p-value
Total no. humans	0.00021	(−0.00024, 0.00066)	0.92	0.359
Humans per house	0.34	(0.23, 0.45)	6,12	<0.001
I_0_ residents	−0.08	(−0.10, −0.061)	−8,17	<0.001
I_0_ biting vectors	0.12	(0.12, 0.13)	28.28	<0.001
Total no. pupae	0.00092	(0.00080, 0.0010)	15.03	<0.001
Patch	−0.00047	(−0.0016, 0.00071)	−0.78	0.436
Intercept	−1.21	(−1.58, −0.83)	−6.31	0.000

I_0_ is the house of residence of the introduced viremic human.

### Epidemic potential

We evaluated the association of entomological surveillance indicators with epidemic potential, averaged over 1000 iterations in which the virus was successfully introduced. All metrics were significantly associated with epidemic potential, but the product of pupal abundance and human density (pupae x humans per house) was a substantially stronger predictor than the others. In particular, pupae x humans per house had over three times the log-likelihood of predicting epidemic potential compared to pupae per house or pupae per humans (Table 3). This indicates a strong interaction between human density and pupal production.

**Table 3:** Univariate association of survey indices with epidemic potential.

*Index*	*Wald χ^2^_(1)_*	Log-likelihood
Pupae x humans per house	1032.3	62.4
Pupae per house	349.5	17.5
Pupae per human	308.3	14.5
BI^1^ x humans	143.4	−18.1
Pupae per (housesx humans)	101.6	−22.7
BI^1^	51.7	−40.4

1 BI = number of vessels with *A. aegypti* aquatic stages/total number of houses.

### Vector control in variable human densities

**Figure 7:**
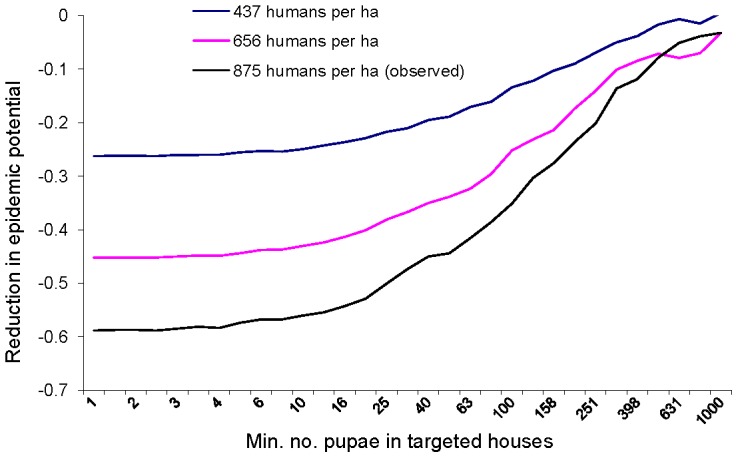
Effect of targeted elimination of pupae in houses with at least X pupae on reducing epidemic potential, compared with non-intervened levels. Human population density is varied relative to census-observed population. Curves represent averages across the 16 study patches. Stochastic variation is greater at highest threshold control values because few patch-surveys had more than 500 pupae in a single house.

We simulated the input of eliminating pupae in houses above a threshold pupal density on epidemic potential under a range of human population densities. This enabled us to eliminate any potential colinearities between human density and vector production in the field data. The substantial differences in the slopes of the curves in Figure 7 indicate that a greater reduction in epidemic potential was achieved at higher human densities as the pupal abundance of containers targeted for control rose (Fig. 7). This re-confirms the interaction between vector production and human density. Targeted source reduction yielded roughly 2.5 times the decrease in epidemic potential when human density was doubled (Fig. 7). However, this magnification occurred when houses with high pupal densities were intervened (Fig. 7). By contrast, increasing the target threshold from 1 to 16 pupae did not significantly affect the epidemic potential in any of the human densities (Fig. 7). These findings indicate that the interaction of vector production and human density was due to the combined impacts of *A. aegypti* super-production on both the spatial aggregation and the overall rate of vector recruitment. If the interaction was caused only by overall vector recruitment, larger differences in the slopes (Fig. 7) would be observed when less productive habitats were targeted.

## Discussion

Understanding how the natural regulation of *A. aegypti* production and human density influence the dynamics of dengue propagation is critical for optimizing vector control programs. Previous modeling studies demonstrate that because most *A. aegypti* are produced in only a few containers, elimination of a small subset of containers is sufficient to drive the dengue R_0_ below 1 [13], [14]. These studies are based on the classical assumption in mosquito borne disease modeling of homogenous mixing of hosts and vectors [19]. This assumption leads to the conclusion that *A. aegypti* 's vectorial capacity decreases as human abundance increases relative to vector abundance [14]. In this paper, through the use of a spatially explicit agent-based model, we were able to relax this assumption in order to assess how observed variations in pupal production and human density reflect variation in the intensity of dengue transmission in a community of houses. Our model re-confirmed the potential for source reduction to substantially reduce dengue by targeting only containers that produce above a threshold number of pupae, but generated surprisingly contrasting results with regards to how pupal production interacts with human density. Rather than an inverse relationship as predicted by the traditional vectorial capacity equation [19], we found that increased human density can favor *R_o_* through both human-to-mosquito and mosquito-to-human transmission. Moreover, when viral introduction was accounted for, human density amplified the effect of *A. aegypti* super-production on dengue risk. By parameterizing vector dynamics with seven seasonal pupal surveys, we show that long-term decreases in vector production can achieve substantially larger reductions in epidemic potential when concentrated in areas of higher human density.

In infectious disease ecology, *super-spreading* occurs when an individual host causes an inordinate number of secondary infections as compared to the majority of hosts [33]. Our model indicates that super-spreading may play a critical role in dengue transmission. Although the large majority of dengue introductions did not generate secondary human infections, 95% of the variation in R_0_ was explained by the frequency of introductions that generated at least 20 secondary human infections. These results are supported by the spatiotemporal clustering of dengue infection at the city-block level [34] and our preliminary data from a pilot study of dengue infection clusters in these same Armenia neighborhoods (Padmanabha et al, in revision). Our model directly links dengue super-spreading to the dominant role of super-productive vessels in vector recruitment. Introductions that resulted in greater than 20 secondary human infections occurred by and large, only when infected mosquitoes were produced in houses among the top 5 percentile of vector production. Moreover, the density of biting *A. aegypti* in the residence of the introduced human infection was the strongest predictor of secondary transmission. This suggests that by concentrating the large majority of the emergent vector population in only a few houses, super-production facilitates human infection of large numbers of mosquitoes, albeit infrequently. Thus, while homogenous mixing models establish that dengue transmission requires a threshold vector density [13], [14], our model mechanistically links the regulation of *A. aegypti* production with the propagation of dengue across houses.

These mechanistic details proved critical to achieving a more complete understanding of the effects of human density on dengue risk. Vector production influenced the transmission cycle through human-to-mosquito transmission. Due to the observed variation in super-productivity across patch surveys (Fig. 2), human-to-mosquito transmission had a much larger impact on variation in secondary infections than mosquito-to-human transmission. In addition to vector production, the introduction of infectious humans also acts through human-to-mosquito transmission. Thus, when we incorporated a likelihood of human introduction into our risk indicator, areas with higher human density had an increased likelihood of having an introduced human infection reside in a house where mosquitoes concentrated, thereby generating an interaction between vector production and human density. Moreover, intra-patch human social interactions caused increased human density to increase the probability that each house, including those where mosquitoes concentrated, received a visit from the introduced infection. This is the reason why increased human density favored human-to-mosquito infection, even though the household size of introduced infections reduced their average contact rate with mosquitoes.

These interactive relationships between human density and vector production may have direct implications for dengue risk assessment and resource allocation for vector control. Previous models assuming homogenous mixing of hosts and vectors predict a threshold value of the index *pupae per person* required for dengue epidemics [14]. We found that the index *pupae x humans per house* had a stronger correlation with epidemic potential than *pupae per person*, a measure that does not account for the human density dependence of both the dengue *R_o_* and viral introduction. Moreover, targeted vector control in areas of high human density may reduce epidemic potential by decreasing the abundance of mosquitoes in areas where dengue is most likely to be introduced. This suggests an opportunity for multi-level targeting of source reduction efforts. Specifically human density could be used to determine in which neighborhoods to focus vector control, and the likelihood of *A. aegypti* super-production could be used to focus efforts within targeted neighborhoods.

Our model reinforces the need to better understand the dynamics of human exposure to mosquitoes outside the house. Given the short lifespan and limited dispersal of urban *A. aegypti*, human social networks are likely to drive the constant re-introduction of dengue into patches. In the absence of data on the type, duration, and location of social contacts that lead to *A. aegypti* exposure, we sought a balance between simplicity and realism in our assumptions regarding the frequency of contact with mosquitoes outside one's home. As such, we excluded major public centers, such as schools, offices or parks, because the residential blocks in our field study were devoid of these. Although these areas are potentially important in transmission dynamics [23], *A. aegypti* contact with visitors is likely to be higher for social contacts that involve household visits, due to *A. aegypti's* endophilic nature and domestic oviposition sites. For example, visits between neighbors, and especially between children [who are more likely to be susceptible to dengue in highly endemic areas], may have more relevance in terms of exposure to *A. aegypti*. Moreover, we have observed that in Colombian cities, working class and marginal areas, such as La Fachada and Las Colinas, have more interactions among neighbors than in affluent areas. This was a major motivation for including a distance dependent component to the intra-patch social contacts in our model. Accordingly, we consider conservative our assumption of daily exposure to *A. aegypti* bites in exactly one other house within the patch. An increase in intra-patch social contacts with *A. aegypti* exposure is likely to heighten the human density dependence of dengue transmission. Future expansion of our work would benefit from an improved understanding of (1) how *A. aegypti* biting habits influence human exposure in non-residential premises and (2) how housing density, age and social class affect the geography and centrality of social networks that involve exposure to *A. aegypti*.

Recently it has been shown that larval environmental conditions, including resource availability and thermal conditions, can affect the vector competence of *Aedes spp* [35], [36]. All things being equal, our study and others [13], [14] indicate that most secondary infections are generated by super-productive habitats. When these habitats are absent explosive transmission was nearly impossible in our model. Because most viral introductions do not generate secondary infections explosive transmission events were critical to the dengue *R_0_*. This suggests that it would be highly beneficial for dengue virus to efficiently infect and disseminate better in mosquitoes produced in super-productive habitats. However, there is a lack of understanding of exactly what eco-physiological conditions are associated with super-productive habitats. Human behavior, particularly emptying frequency and water usage, unquestionably plays an important role [7], [8].It is also conceivable that because super-productive containers necessarily have large L4 cohorts, which can exert significant competitive pressure on one another [9],the physiology of adult mosquitoes has particular adaptations to resource poor environments. Mild resource competition in the larval environment has been shown to favor dengue infection and dissemination in *A. aegypti* [36]. Our results suggest that this finding could be an outcome of viral adaptation to mosquitoes produced in super-productive vessels. Furthermore, our model can be used to explore the eco-epidemiological implications of such evolution. We speculate that it would intensify the interactive effect between super-production and human density in favoring dengue risk.

In summary, we found that variation in *A. aegypti* production across socio-ecologically similar urban patches can generate large variation in secondary transmission and the epidemic potential of dengue, with human population density magnifying these effects. Our results suggest that *super-spreading* plays an important role in dengue transmission and occurs when a viremic human is bitten by a large number of mosquitoes that were produced in a super-productive vessel. Human density, in turn, can potentiate the epidemiological significance of super-productive *A. aegypti* habitats. These results re-affirm the importance of spatial heterogeneity in fine-scale dengue dynamics [37]. Moreover, because both human density and the frequency of *A. aegypti* spuper-production may vary widely within rapidly urbanizing developing countries, our results may be useful for stratifying risk. By contrast, while the dengue system is theoretically very sensitive to *A. aegypti* survival and biting rates [38], [39], there is little evidence to suggest that either of these processes will significantly vary across areas in the same city with similar climatic conditions. We suggest that by mechanistically evaluating the epidemiological impacts of observed socio-ecological variation, further modeling studies can contribute to the development of a comprehensive framework for stratifying epidemic risk and optimizing dengue prevention resources.

## Supporting Information

Text S1 **This section describes how we modeled the rate of mosquito recruitment in houses with missing entomological data.**
(DOC)

Table S1 **Summary of parameter descriptions, values/distributions and sources.**
(DOC)
