# Supplementary material for: The Interactive Roles of Aedes aegypti Super-Production and Human Density in Dengue Transmission
Source: PLoS Negl Trop Dis. 2012 Aug 28;6(8):e1799. doi: 10.1371/journal.pntd.0001799 (PMC3429384; doi:10.1371/journal.pntd.0001799)
Supplement: Text S1 — This section describes how we modeled the rate of mosquito recruitment in houses with missing entomological data. (DOC) [file pntd.0001799.s001.doc]

Supplementary text

*Simulating vector production in houses with missing data*

To fill in missing data for simulations of each patch-survey, we used a Bernoulli process to assign an infestation status of positive or negative for *A. aegypti* aquatic stages to each uninspected premise. A number of pupae were randomly assigned to each premise given a positive status, based on the step-function generated by the frequency distribution of pupae that was observed across all positive containers identified in each respective neighborhood-survey period. For premises with at least four inspections, the Bernoulli probability used to determine infestation status in a missing survey was the fraction of inspections of that premise in which a positive container was observed. For those premises with three inspections or fewer, the Bernoulli probability of infestation was the overall fraction of house-visits for each neighborhood with at least one container positive for *A. aegypti*. This algorithm for filling in missing data was chosen to balance the tradeoff between conserving the distribution of production that was observed across premises and the propensity of certain premises to be infested with *A. aegypti* due to their particular social and ecological conditions.
