# Supplementary material for: The Interactive Roles of Aedes aegypti Super-Production and Human Density in Dengue Transmission
Source: PLoS Negl Trop Dis. 2012 Aug 28;6(8):e1799. doi: 10.1371/journal.pntd.0001799 (PMC3429384; doi:10.1371/journal.pntd.0001799)
Supplement: Table S1 — Summary of parameter descriptions, values/distributions and sources. (DOC) [file pntd.0001799.s002.doc]

Table S1. Model parameters

| Symbol | Description | Value | Distribution | Source |
| --- | --- | --- | --- | --- |
| t_h_ | Incubation time of Dengue fever in humans |  | Uniform[4.5,7] days | [23] |
| d_h_ | Time that a person remains in the infectious state |  | Uniform[3,5] days | [37, S1] |
| P_inf_h | Probability that a person bitten by an infectious mosquito will become infected | 0.75 |  | [37, S1] |
| pIndep | Bernoulli probability distance-independent component for choosing homes for the permanent set of contacts | 0.2 | +/- 10% | Specified |
| Cd | Bernoulli probability constant for choosing homes for the permanent set of contacts | 0.2 | +/- 10% | specified |
| visitW | Weight assigned to probability that mosquito bites visitor | 0.2 | +/- 10% | specified |
| p_intro_ | Probability of infection introduction on the first stable day of the simulation | 0.001 |  | specified |
| nVisits | Number of visits that a person makes each day | 1 day^-1^ |  | specified |
| minBiteLogAge | Minimum age to record the mosquito bite rate | 15 days |  | specified |
| t_m_ | Incubation time of Dengue virus in mosquitoes |  | Uniform[10, 15] days | [13, 23, 37, S1, S2] |
| π | Maximum number of bites a mosquito can inflict each day | 10 bites/day | +/- 10% | Calibrated to [30] |
| p_full_ | Bernoulli probability of a mosquito becoming full at each bite |  | Uniform[0.25,5] | Calibrated to [30] |
| β_mos_ | Probability that each mosquito that bites a viremic person will become infected | 0.75 |  | [13,23,37,S1] |
| σ_mos_ | Standard deviation of the Gaussian flight distribution |  | Uniform[2.3,3.5] | Calibrated to [30] |
| s_v_ | Probability of mosquito survival each day | 0.84 day^-1^ | Beta[6,6]*(0.89–-0.79) + 0.79 | [13,38] |
| p_female_ | Probability of a produced pupa to be female | 0.5 |  | Specified |
| pup | # days until pupal emergence in Armenia (water temperature 21–-22°C) | 2.7 days |  | field measured |
| s_pup_ | Fraction of pupae that survive | 0.94 day^-1^ |  | Field measured |
| refr | Post-emergence pre-mating refractory period in which mosquito does not host seek in Armenia (ambient temperature 20–-24°C) | 3 day |  | [29] |
| disp_non_biting | # days after emergence that mosquito may disperse | 2 days |  | Specified |

Supplemental references

S1. Luz PM, Codeco CT, Massad E, Struchiner CJ (2003) Uncertainties regarding dengue modeling in Rio de Janeiro, Brazil. Memorias Do Instituto Oswaldo Cruz 98: 871-878.

S2. Watts DM, Burke DS, Harrison BA, Whitmire RE, Nisalak A (1987) Effect of temperature on the vector efficiency of Aedes aegypti for dengue 2 virus. Am J Trop Med Hyg 36: 143-152.
